# Diagnostic performance of shear wave elastography and diffusion-weighted magnetic resonance imaging in cervical lymph nodes: a comparative study

**DOI:** 10.3906/sag-2012-62

**Published:** 2021-07-31

**Authors:** Veli Süha ÖZTÜRK, Ersen ERTEKİN

**Affiliations:** 1 Department of Radiology, Faculty of Medicine, Adnan Menderes University, Aydın, Turkey; 2 Department of Radiology, Salihli State Hospital, Manisa, Turkey

**Keywords:** Lymph nodes, ultrasound, sonoelastography, diffusion weighted MRI

## Abstract

**Background/aim:**

To investigate the potential diagnostic value of point shear wave elastography (pSWE) and the contribution of concurrent diffusion weighted magnetic resonance imaging (DW-MRI) to diagnostic performance in patients with cervical lymphadenopathy.

**Materials and methods:**

This cross-sectional study included 116 cervical lymph nodes of 94 patients. All lymph nodes were evaluated before the treatment or histopathological sampling. Gray scale ultrasonographic features, elastographic stiffness and apparent diffusion coefficient (ADC) values were measured and recorded. Lymph nodes were divided into benign and malignant groups with histopathological findings.

**Results:**

Short axis measurement, axis ratio, hilum morphology, vascularization patterns, pSWE, and ADC values were the most significant parameters in logistic regression tests. The median stiffness of malignant nodes was higher and the mean ADC values were lower than others. Also, lymphoproliferative disorders had the lowest ADC values (p < 0.001). Area under the curve values for pSWE and DW-MRI were 0.852 [95% confidence interval (CI), 0.779–0.925], 0.790 (95% CI, 0.695–0.885), respectively. The accuracy rate increased from 79.3% to 85.3% when the pSWE was combined with the ultrasonography (US).

**Conclusion:**

The use of pSWE combined with conventional US will reduce the number of biopsies and may be sufficient to differentiate the lymph nodes.

## 1. Introduction

Cervical lymph nodes (LNs) may grow and become pathological due to benign and malignant reasons. This is important in predicting prognosis, staging and determining the treatment method in patients with malignancy [ 1]. Although many features of LNs can be evaluated by ultrasonography (US), these parameters alone are not sufficient to differentiate etiologies [2,3 ] and the elastography US, a new tool, can help with this [ 4]. Shear wave elastography (SWE), through which the images are produced with acoustic radiation force impulse (ARFI), allows quantitative measurement of lesion stiffness [5]. SWE has been used to measure tissue stiffness in many organs and tissues and was considered successful in distinguishing benign/malignant lesions [6,7 ]. Another imaging method frequently used for LNs evaluation is magnetic resonance imaging (MRI). Conventional MRI sequences are very helpful in detecting lesions. But diffusion-weighted MRI (DW-MRI) is a functional method showing cellularity which is very useful in the differential diagnosis of benign/malignant LNs and the diagnosis of lymphoproliferative diseases [8–10]. Additionally, diffusion imaging stands out as a quantitative imaging method.

Invasive methods such as biopsy have been used as the gold standard in the diagnosis of pathological lymph nodes in routine practice [11]. Although fine needle aspiration biopsy (FNAB) is frequently used with a low complication risk, it has not been able to provide adequate diagnostic performance in some cases, especially in lymphoproliferative diseases, as it is an evaluator and practitioner-dependent method [12]. Increasing the success of diagnostic tests and using them together may also be beneficial in terms of avoiding invasive methods, selecting the patients who need biopsy accurately and reducing the number of interventions.

We could not find any study evaluating and comparing US, SWE and DW-MRI findings for LNs together. The present study aimed to investigate the potential diagnostic value of SWE and the possible contribution of simultaneous DW-MRI to sonographic diagnostic performance in patients with pathological cervical LNs and to develop a diagnostic algorithm.

## 2. Materials and methods

### 2.1. Study design and patient population

This cross-sectional study was conducted in accordance with the principles of the Declaration of Helsinki. Procedures were thoroughly explained to all patients and their informed consent was obtained. The Ministry of Health, Medicines and Medical Devices Agency and the clinical research Ethics Committee of our hospital approved the protocol of the study (decision no: 15.03.2018/2).

One hundred and sixteen LNs of 94 patients aged between 18 and 87 years who applied to our institution for neck US examination between March 2018 and September 2019 were included in the study. Gray scale US, Color Doppler US (CDUS) and SWE examinations were performed in all cases. Patients aged 18 years and over with cervical lymph nodes with at least one of the sonographic pathological features discussed in the article were included in the study. At most two pathological lymph nodes of the same patient could be included in the study. All lesions were evaluated before treatment or biopsy and all had pathological results. Lesions smaller than 5 mm were excluded due to the fixed region of interest (ROI) size. Those with cystic-necrotic changes or calcifications in more than half of the lymph nodes were also excluded. DW-MRI examination was completed in 85 LNs of 68 patients. Eleven patients with cystic necrotic changes in ultrasonography, seven with an unstable general condition, four with MRI contraindications and four with gross artifacts in examinations were excluded only from diffusion imaging. If there was any cystic-necrotic change on US, MRI was not performed regardless of the size. The results of all sonographic examinations were recorded on the radiological imaging and archive system. All US and MRI examinations were obtained by one radiologist with three years of experience in general radiology. All recorded US, CDUS, SWE images and magnetic resonance (MR) images were reevaluated together with the radiologist who had 15 years of general radiology experience in the data imaging system and concluded with consensus.

### 2.2. Gray-scale and Doppler ultrasonography

The patients were placed in the supine position on the examination table and the neck area was stretched with a pillow placed under the neck. Gray scale and Doppler US examination were obtained using the Samsung Medison RS80A Prestige (Samsung Medison Co. Ltd., Seoul, Korea) ultrasound system, high-resolution linear probe (L3–12A) at a frequency range of 3–12 MHz. Lymph node (LN) locations were classified from one to six based on the American Joint Committee on Cancer (AJCC) lymph node classification system [13]. Short and long axis diameter, axis ratio (short axis/long axis), and border; the presence of echogenic hilum, heterogeneity, calcification; cystic-necrotic changes and intranodal reticulation were evaluated. Parameters in the neck application of our device were used for all patients without changing any conditions. CDUS and power Doppler US techniques were used as the Doppler imaging method. Vascularization patterns were evaluated and divided into three subgroups: avascular (type I), hilar/central (type II) and peripheral and/or mixed (type III).

### 2.3. Shear wave Eeastography

**Figure 1:**
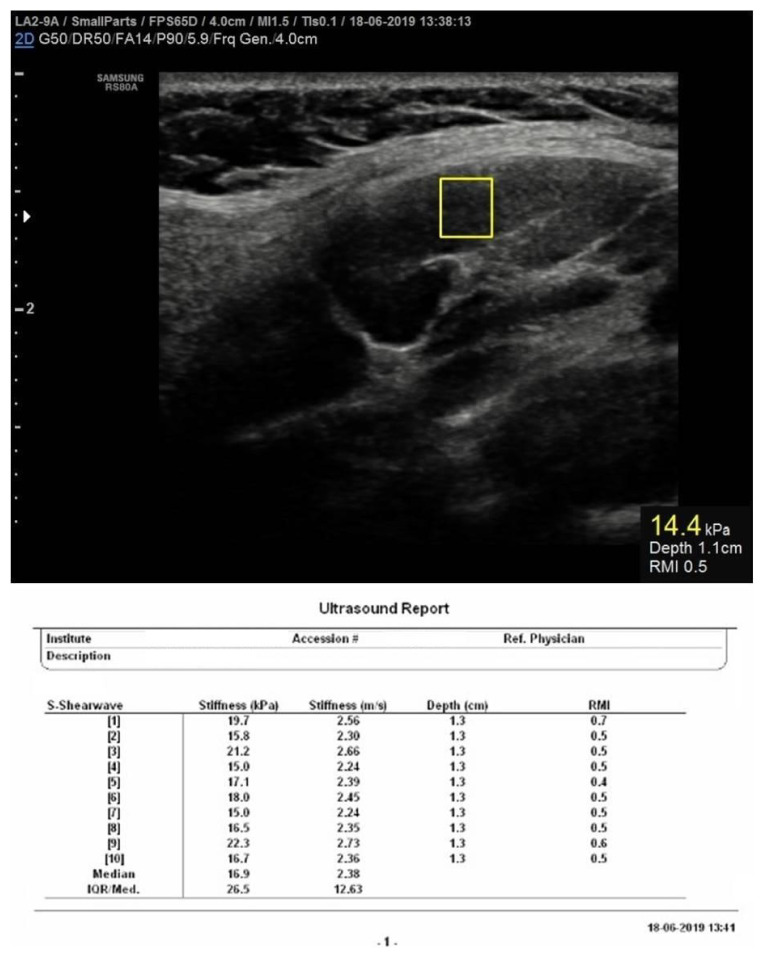
Point shear wave elastography measurement method.

Images were produced using the S-Shear Wave Elastography (Samsung Medison Co. Ltd., Seoul, Korea) application, working with the Samsung Medison RS80A Prestige (Samsung Medison Co. Ltd., Seoul, Korea) ultrasound system and with a frequency range of 2–9 MHz broadband linear probe (LA2-9A). The application worked with ARFI based point shear wave elastography (pSWE) technique. The probe was placed vertically on the skin without applying pressure. The LNs were placed in the middle of the examination area. A fixed size (0.5 cm × 0.5 cm) sampling box (ROI), which can be measured reliably, was placed on the LN in the 0.5–3 cm range depth when the case was completely immobile. ROI was attempted to be placed in the cortex by avoiding cystic - necrotic areas, calcification and hilum as much as possible and without going beyond the LN borders. Velocity (m/s), stiffness (kilo Pascal - kPa) values, reliability measurement index (RMI) and interquartile range/median ratio (IQR/M) of the sampled area were demonstrated on the results. All values were recorded as a screenshot after the examination. Quantitative median stiffness values were calculated after at least 10 measurements with an RMI value of 0.4 and above in all lesions [14]. IQR/M was aimed to be kept below 30% in all LNs [14–16] (Figure 1).

### 2.4. Diffusion weighted imaging

Images were obtained with a 1.5 Tesla MR device (Philips Achieva, Philips Medical Systems, Nederland BV) using a 16-channel neurovascular coil. Respiratory triggering was used for each examination. DW-MRI was obtained by applying diffusion sensitive gradients in an axial plan, single-shot echo-planar sequence, three directions (x, y, z) and three different b values (b = 0, b = 200, b = 800 mm^2^/s). Images were transferred to a different software program (Myrian, Imoios, Montpellier, France) and apparent diffusion coefficient (ADC) measurements were automatically calculated from maps created according to b = 0 and b = 800 values after drawing ROI in an axial plan. Measurements were based on an entire LN by avoiding its borders. The mean ADC value was recorded from the automatically generated histogram analysis table. The smallest LN size was 7 mm (mean 14.3 ± 7.1). All measurements were completed within 24 h to ensure that the evaluations were performed in the same lesion. The order of examination was gray-scale US, pSWE and ADC measurement. The second researcher confirmed all measurements within the next six hours, with ADC measurement first and then pSWE. The localization of LNs was reviewed by two radiologists. Size and shape parameters were used as confirmatory during selection. FNAB-based histopathological examination was performed within one month at the latest. The times of excisional biopsy were not evaluated. The skin projection was marked before excision if multiple pathological lymph nodes were present.

### 2.5. Pathological diagnosis

All LNs were analyzed by the pathology unit of our hospital and cytological and/or histopathological diagnosis was carried out after the radiological assessment. The histopathology result was used for diagnosis when both were available.

### 2.6. Data analysis

Data were analyzed using the SPSS v.22 software package program (SPSS Inc.). Data normality was evaluated with the Shapiro–Wilk test. Continuous variables that conform to the normal distribution were presented as mean ± standard deviation and those did not fit as median (minimum-maximum). An independent sampling t-test was used if numerical data were normally distributed. The Mann–Whitney U test was used for the nonnormally distributed data. Categorical variables were evaluated using Pearson’s chi-square or Fisher’s exact chi-square tests. Sensitivity, specificity, positive predictive value (PPV); negative predictive value (NPV) and accuracy were calculated for each predictor. The area under the curve in 95% confidence interval was calculated by receiver operating characteristic (ROC) analysis in continuous data. Cut-off values were obtained from the ROC curve using Youden’s index. All parameters were used together to differentiate benign and malignant LNs in the logistic regression (LR) tests. The relationships of the findings with the result were demonstrated with their relative risk values and diagnostic performances were presented in significant models. A p value <0.05 was significant.

## 3. Results

One hundred and sixteen LNs of 94 patients were included in the study. The mean age was 51 (±17) and 61 (59%) of the patients were female. Fifty-three LNs were benign (29 benign cytology, seven reactive lymphoid hyperplasia, ten granulomatous lymphadenitis, three acute lymphadenitis, two toxoplasma lymphadenitis, one tularemia and one Kikuchi disease) and 63 were malignant (40 metastases and twenty three lymphomas). Metastases were classified as squamous cell carcinoma (n = 19), adenocarcinoma (n = 12), papillary thyroid carcinoma (n = 5); metastasis of unknown origin (n = 3) and breast cancer metastasis (n = 1). Fifty-four percent were diagnosed with FNAB, 3% with tru-cut biopsy and 43% with excisional biopsy. Follow-up US examinations were performed for all lesions diagnosed as benign. The mean sonographic follow-up time was 9 (± 5) months. None of the benign lesions developed any size increase or sonographic findings suggestive of malignancy. According to the AJCC classification system, 49.1% of benign nodes were at level II while 44.4% of malignant nodes were at level V.

The dimension of the short-axis was between 5 and 28 mm (median 9 mm) in benign and between 5 and 53 mm (median 15 mm) in malignant LNs. Axis ratio (short axis/long axis) of the benign and malignant LNs were between 0.23–1 (median 0.5) and 0.33–1 (median 0.71), respectively. The diagnostic performances of the threshold values obtained from ROC analysis and detailed results of all parameters were presented in Table 1. Area under the curve (AUC) values for short axis and axis ratio were 0.750 [95% confidence interval (CI), 0.661–840] and 0.762 (95% CI, 0.675–0.849), respectively. There were significant differences between the groups. The irregular border and loss of echogenic hilum, presence of cystic-necrotic changes and peripheral/mixed vascularization were other significant features in malignant LNs (p < 0.05). There were no significant differences between the groups in the evaluation of the long axis, trabecular pattern and cortical calcification (p > 0.05).

Stiffness values were between 7.5 and 102 kPa (median 14.9 kPa) in benign and between 10.9 and 185.3 kPa in malignant LNs (median 48.8 kPa). Tissue stiffness values were significantly higher in the malignant group (p < 0.001). The cut-off value for elasticity was determined as 30 kPa with the ROC curve. AUC value for pSWE was 0.852 (95% CI, 0.779–0.925). The sensitivity and negative predictive value were 100% when the threshold value was accepted as 10 kPa. Additionally, benign and malignant LNs were divided into two subgroups for a detailed analysis (Table 2). The granulomatous-inflammatory group had significantly higher stiffness values than the reactive group and the metastatic group had significantly higher stiffness values than others (p < 0.05).

The mean ADC values were 1.291 ± 0.318 × 10^−3^ mm^2^/s in benign and were 0.951 ± 0.319 × 10^−3^ mm^2^/s in malignant groups. ADC values were significantly lower in the malignant lesions (p < 0.001). The AUC value for ADC was 0.790 (95% CI, 0.695–0.885). The mean ADC value of the primary lymphoid tumor group was significantly lower than other subgroups (p < 0.001) (Table 2).

**Figure 2:**
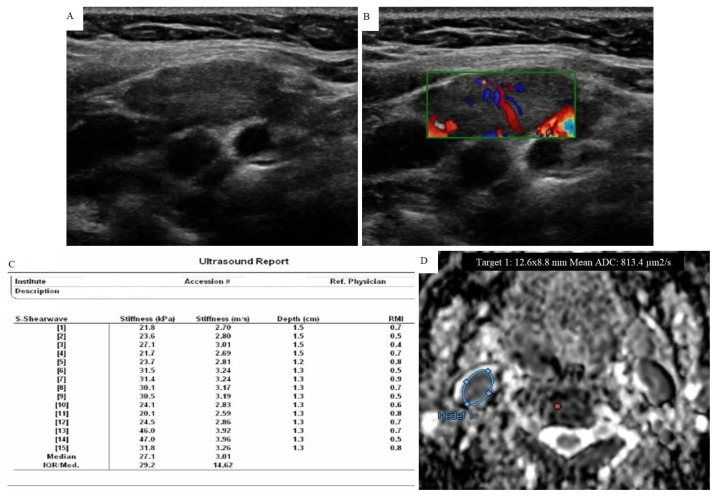
A 48-year-old woman with benign cytology diagnosed with FNAB. A) There was a lymph node that newly developed in follow-up with 13 × 9 mm size, well-circumscribed, smooth border and ovoid-shaped at the level II on the right side of neck, in the case with a history of thyroid papillary cancer on the same side. However, normal fatty hilum could not be identified. B) Doppler US examination showed type II vascularization. C) pSWE parameters are shown in the table. The median stiffness value was found as 27.1 kPa (3.01 m/s) according to the elasticity measurement. D) The ADC map shows the area where the measurement was made. The average ADC value was found to be 0.813 × 10^−3^ mm^2^/s. It was a remarkable example with low ADC value and showed that MRI could be confusing with unnecessary use.

**Figure 3:**
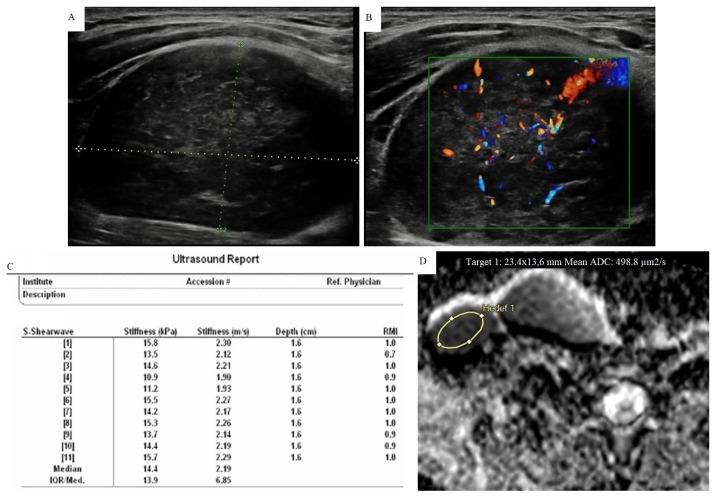
An 18-year-old woman diagnosed as mixed cellular Hodgkin lymphoma after lymph node excision. A) In the case with multiple lymph nodes in the neck, the largest was at level 5 on the right with a 48 × 31 mm. It showed heterogeneous internal structure and trabecular pattern. Also note that normal echogenic hilum was absent. B) Type III mixed vascularization was seen in CDUS. C) pSWE parameters are shown in a table. The median stiffness value was found as 14.4 kPa (2.19 m/s) according to the elasticity measurement. D) The ADC map shows the area where the measurement was made. The average ADC value is found to be 0.498 × 10^−3^ mm^2^/s. Nodes in lymphoproliferative diseases may be softer than expected. However, the diagnostic success of ADC measurement in this group was very high.

**Figure 4:**
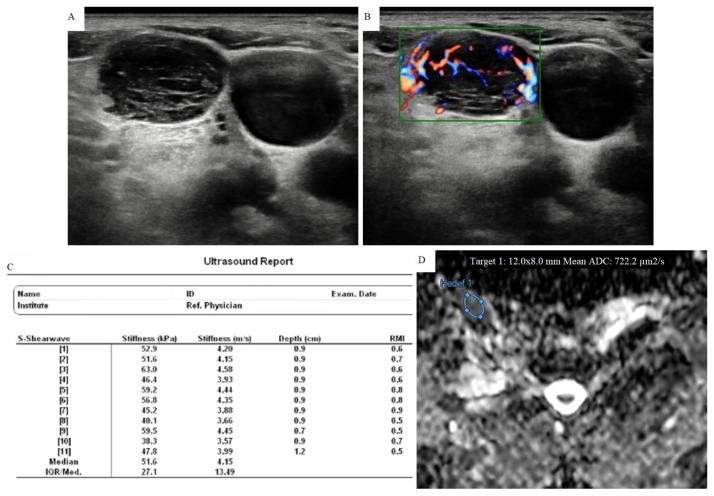
A 61-year-old woman with squamous cell carcinoma metastasis diagnosed with FNAB. A) In the case with a history of lung cancer, 17 × 12 mm sized lymph node was developed in follow-up in the level V on the right side. Lymph node had smooth contour, round shaped, fairly heterogeneous parenchyma and normal echogenic hilus could not be seen. B) Type III mixed vascularization pattern was observed in CDUS. C) pSWE parameters are shown in a table. The median stiffness value was found as 51.6 kPa (4.15 m/s) according to the elasticity measurement. D) The ADC map shows the area where the measurement was made. The average ADC value was found to be 0.722 × 10^−3^ mm^2^/s. It was in the malignant group with both elasticity and ADC values.

Nine of 11 parameters, which were reevaluated with the LR test, were significant for benign-malignant differentiation (Table 3). Only six parameters (short axis measurement, axis ratio, absence of echogenic hilum; CDUS classification, stiffness and ADC value) were highly significant and related to the result at a 95% confidence interval. Only the gray-scale ultrasonography findings were reevaluated together in Model 1. Significant parameters in this model were the short axis measurement, axis ratio and hilum appearance. In model 2, CDUS findings were added to the previous model and the value of accuracy increased from 75.9% to 79.3%. Model 3 demonstrated a significant increase in diagnostic success by adding pSWE to US. The sensitivity, specificity and accuracy values increased to 87.3%, 83%, and 85.3%, respectively. The US findings and ADC values were evaluated together in Model 4 and had an accuracy of 72.9%. In Model 5, ADC data were added to all findings. The overall diagnostic accuracy increased from 85.3% to 85.9% in the comparison of Models 3 and 5 were compared and the difference was not significant (Figures 2a–2d, 3a–3d, 4a–4d).

## 4. Discussion

Although radiological methods are frequently used to detect cervical lymphadenopathies, the reliability of diagnostic parameters (size, shape, internal structure, vascularization, etc.) is still discussed [ 3,17–1 9]. Histopathological evaluation is the gold standard diagnostic method in LNs and mostly requires excision [ 20]. So, different methods are still studied for the diagnosis of LNs. We believe that effective use of all radiological methods will increase diagnostic accuracy and prevent unnecessary invasive procedures. Therefore, we investigated the effects of the B-mode US, CDUS, pSWE and DW-MRI methods on the differential diagnosis of LNs and aimed to develop an effective algorithm with these methods. We proved that the most reliable parameters were stiffness, ADC value, vascularization pattern, hilum appearance, axis ratio and short axis measurement, respectively. Our LR test results showed that when pSWE was added to B-mode US and CDUS, the sensitivity, specificity and accuracy rates increased from 84.1% to 87.3%, from 73.6% to 83% and from 79.3% to 85.3%, respectively. These findings were similar to those in the meta-analysis by Suh et al. and showed that SWE was an acceptable and useful imaging method in benign/malignant lymph node differentiation [21]. The stiffness value alone was more successful than Models 1, 2 and 4 with an accuracy rate of 81%. When DW-MRI was added to all data, sensitivity, specificity and accuracy were 90.9%, 80.5%, and 85.9%, respectively and there was no significant increase. The decreased accuracy rate in Model 4 suggested that using ADC and US together without SWE can be confusing the diagnosis. Hence, pSWE, whether alone or in combination with conventional US and DW-MRI; is regarded as the most important parameter in the evaluation of lymph nodes.

In the sonographic literature, the most frequently used parameter for the detection of pathological LN is the short axis. Cut-off values ranging from 5–10 mm were used in the benign/malignant differentiation in previous studies with sensitivity ranging from 70%–80% for short axis [22–24]. Similarly, we preferred to use a cut-off value of 9.5 mm. Short axis measurement was the most sensitive B-mode imaging finding in our study. Lyshchick et al. showed 65% accuracy by using an 8 mm cut-off point and this study had the closest value to ours [25]. The shape and axis ratio are qualitative and quantitative parameters that can be handled together. LN shape is determined by the axis ratio (short-axis/long axis). In the literature, cut-off values ranged from 0.5–0.6 for axis ratio and sensitivity and specificity ranged between 46%–75% and 56%–88% for these values, respectively. These values were compatible with our findings [25–28]. So, we may assume that malignant LNs often have a round shape. However, benign submandibular and parotid nodes may also be round [29]. Similar to our study, 84%–92% of benign LNs had echogenic hilum and 76%–96% of malignant had lost hilum echogenicity [17, 25]. Although fatty hilum is present in the normal anatomy of LNs, it is possible to see normal structure in early metastatic disease if the medullar lymphatic sinuses are still intact [ 30]. This parameter with high sensitivity and low specificity should not be used alone in the characterization of lesions. Another important parameter was the vascularization type of the LNs and all data obtained with CDUS were compatible with the literature. Normal and reactive nodes are often avascular or show hilar/central vascularization while metastatic nodes often show peripheral and/or mixed vascularization [31–33].

Although conventional US data are important, new methods to increase diagnostic accuracy are investigated and the recently developed elastography US and diffusion MRI play a major role in this regard. Using a cut-off value of 30 kPa (3.16 m/s), we obtained 79.4% and 83.9% for sensitivity and specificity, respectively (p < 0.001). A study by Fujiwara et al., which was published in 2013 and used the pSWE technique, found the shear wave velocities of metastatic nodes to be higher than reactive ones. Also, they reached 95% sensitivity and 81.8% specificity at a 1.9 m/s cut-off value [34]. In another study that used the same technique with 123 cases, Meng et al. reported 82.9% sensitivity and 93.1% specificity values for the cut-off point of 2.595 m/s [35]. Similar to our study, tissue stiffness was higher in malignant LNs than others, but different results were observed between studies for cut-off values and accuracy rates. This might have mainly resulted from the differences between patient populations and the various devices used in the studies. We found that the stiffness values of the granulomatous-inflammatory group overlapped with the malignant group. Seven of the nine false-positive nodes in the pSWE examination included granulomatous-inflammatory groups (five granulomatous lymphadenitis, one tularemia and one acute lymphadenitis cases) in our study. High false positivity in granulomatous diseases may be associated with fibrosis, calcifications and adjacent tissue adhesions observed in the granulomatous reaction [36]. When 17 lymph nodes in this group were excluded and the differentiation of benign-malignant lymph nodes was reevaluated, the sensitivity was 83% and specificity 92% for the 26.7 kPa threshold value. If the number of patients in this group decreases, the cut-off values will decrease and the diagnostic accuracy will increase. Unlike the granulomatous-inflammatory group, LNs of primary lymphoid tumors tend to be softer than metastatic ones due to the absence of desmoplastic/fibrotic reaction [37, 38]. In a study involving metastatic and lymphomatous nodes, chronic lymphocytic leukemia/small lymphocytic lymphoma patients had the lowest mean shear wave velocities [ 39]. We believe that this is another reason why our study had a relatively low sensitivity. Six of 13 false-negative results were in primary lymphoid tumor groups and this supported our hypothesis. In line with the literature, pSWE was the most useful parameter in distinguishing between benign and malignant LN [34, 35, 40]. Similar to the literature, we found that pSWE was more successful than US and DW-MRI even when it was used alone.

Mean ADC values of malignant nodes were significantly lower than benign nodes. We were able to differentiate malignant nodes at a cut-off value of 1.04 × 10^−3^ mm^2^/s with 51.8% sensitivity and 75.6% specificity. Although there was little variation for cut-off values between studies, our study had a lower diagnostic performance [ 41,42]. We attributed our low sensitivity to two reasons. The granulomatous-inflammatory and metastatic LNs had close ADC values and our study had a much higher amount of granulomatous-inflammatory LNs than others. The benign group consisted of homogeneous LNs mostly diagnosed with reactive hyperplasia in the studies by Perrone et al. and de Bondt et al. [ 41,42]. This may have caused an increased sensitivity. Contrary to this, we had a much more heterogeneous sample. We diagnosed the lymphomatous group with 92.6% sensitivity and 76.5% specificity at a 0.819 × 10^−3^ mm^2^/s threshold value when we excluded the granulomatous group and evaluated malignant lesions only. These values were similar to those in the study of King et al. [ 43].

We showed 100% specificity in terms of the irregular border, which is a marker of extracapsular invasion. Similarly, cystic/necrotic changes had 94.3% specificity. These data were similar to the literature. As they have high specificity, biopsy should be strongly recommended if cystic /necrotic change or irregular border is present [25,39, 44]. Ultrasound is commonly used together with FNAB which has high sensitivity and specificity [45]. Although it is a reliable and sensitive method, FNAB, which is an invasive approach, can cause confusing results such as nondiagnostic cytology due to the heterogeneous structure of the lymph node. Therefore, the search for an alternative method to FNAB continues and SWE becomes more prominent. Another important issue to consider is when a biopsy can be avoided. In this case, NPV of diagnostic parameters gains importance. pSWE had the highest NPV value in our study. We reached the highest negative predictive value (100%) when the threshold value was 10 kPa. This is important while selecting patients for FNAB. If there are low suspicious findings in the lymph node, but the stiffness value is 10 kPa and below, FNAB may not be performed on this lymph node and can be followed up with the results of our study.

Similar to the literature, we aimed to evaluate the US, SWE and DW-MRI findings together to predict a stepped imaging system and produce a diagnostic algorithm. Gray scale US, CDUS and SWE can be a critical problem-solver in characterizing LNs and choosing the FNAB when they are used together. There is no need to use diagnostic DW-MRI instead of the US which is a cheaper and more easily accessible and applicable method. However, DW-MRI should be preferred with very high sensitivity in case of a suspicion of lymphoproliferative disease or primary head and neck malignancy. DW-MRI may be a second step examination if the US provides insufficient information. The high negative predictivity of SWE should be regarded when it is difficult to make a decision.

Our study had some limitations. We had heterogeneous patient groups and used different sampling methods for pathological diagnosis. Nearly half of the cases were only diagnosed cytologically by FNAB and the sample size was relatively small. The fixed ROI size was another limitation that could not be changed. Lack of inter/intraobserver reliability assessments and the absence of b = 1000 s/mm^2^ and simultaneous conventional MRI examination were other limitations. The researcher who measured the ADC was aware of the pSWE values. Although this was an inevitable source of bias, it was tried to be overcome by the control mechanism provided by the second researcher.

In conclusion, the combination of point shear wave elastography and conventional ultrasonography may be used in the differentiation of benign lymph nodes from metastatic lymph nodes and significantly reduce the number of biopsies. DW-MRI should only be preferred in suspected cases and/or the suspicion of the lymphoproliferative disease. Findings should be supported by further prospective/multicenter studies with similar SWE and MRI protocols in large case series.

## Figures and Tables

**Table 1:** All evaluated features and statistical results of lymph nodes.

LN features	Cut-off /clasification	Benign (n = 53)	Malign (n = 63)	Sensitivity (%)	Specificity (%)	PPV (%)	NPV (%)	Accuracy (%)	p value
**Short axis (mm)**	9.5 <	28	10	84.1	52.8	67.9	73.7	68.8	< 0.001^a^
	9.5 ≥	25	53						
**Axis ratio**	0.54 <	34	14	77.8	64.2	72.1	70.8	71.6	< 0.001^a^
	0.54 ≥	19	49						
**Border**	Smooth	41	40	17.5	100	100	49.5	55.2	= 0.006^b^
	Lobulated	12	12						
	Irregular	-	11						
**Parenchyma**	Homogeneous	43	40	36.5	81.1	69.7	51.8	56.9	= 0.036^b^
	Heterogeneous	10	23						
**Hilum**	Normal	30	9	85.7	56.6	70.1	76.9	72.4	< 0.001^b^
	Abnormal	23	54						
**Calcification**	(−)	45	56						= 0.52^b^
	(+)	8	7						
**Cystic-necrotic**	(−)	51	53	15.9	96.2	83.3	49	52.6	= 0.033^b^
	(+)	2	10						
**Trabecular pattern**	(−)	52	56						= 0.069^b^
	(+)	1	7						
CDUS type*	Type 1	19	10	71.4	71.7	75	67.9	71.6	< 0.001^b^
	Type 2	19	8						
	Type 3	15	45						
pSWE* **(kPa)**	≤ 30	44	13	79.4	83	84.7	77.2	81	< 0.001^a^
	> 30	9	50						
		**Benign (n = 41)**	**Malign (n = 44)**						
ADC* **(10^−3^ mm^2^/s)**	> 1.04	31	18	51.8	75.6	72.2	63.3	67.1	< 0.001^c^
	≤ 1.04	10	26						

ADC: Apparent Diffusion Coefficient, CDUS: Color Doppler Ultrasound, kPa: Kilo Pascal, LN: Lymph Node, PPV: Positive Predictive Value, NPV: Negative Predictive Value, pSWE: Point Shear Wave Elastography. Significance tests used:

a Mann–Whitney U test,

b chi-square test and

c t-test.

**Table 2:** Distribution of stiffness and ADC values within subgroups.

	LN subgroup	Mean kPa^*^	Cut-off (kPa)	Sensitivity (%)	Specificity (%)	PPV (%)	NPV (%)	Accuracy (%)	AUC	p value
pSWE* **(kPa)**	Reactive (n = 36)	16.3	26	47.1	88.9	66.7	78	75.5	0.702	= 0.019^a^
	Granulomatous (n = 17)	37.7								
	Lymphoid (n = 23)	48.1	50	60	78.3	82.8	52.9	66.7	0.680	= 0.018^a^
	Metastatic (n = 40)	70.2								
	**LN subgroup**	Mean ADC*	**Cut-off (mm** **^2^** **/s)**	**Sensitivity (%)**	**Specificity (%)**	**PPV (%)**	**NPV (%)**	**Accuracy (%)**	**AUC**	**p value**
ADC* **(×10^−3^mm^2^/s)**	Reactive (n = 28)	1.337	-	-	-	-	-	-	-	> 0.05^b^
	Granulomatous (n = 13)	1.195								
	Lymphoid (n = 17)	0.729	0.819	92,6	76.5	86.2	92.6	86.4	0,878	< 0.001^b^
	Metastatic (n = 27)	1.092								

ADC: Apparent Diffusion Coefficient, AUC: Area Under the ROC Curve, kPa: Kilo Pascal, LN: Lymph Node, NPV: Negative Predictive Value, PPV: Positive Predictive Value, pSWE: Point Shear Wave Elastography.

Significance tests used:

a Mann–Whitney U test and

b t-test.

**Table 3:** Logistic regression test results.

	Model 1 (n = 116)		Model 2 (n = 116)		Model 3 (n = 116)		Model 4 (n = 85)		Model 5 (n = 85)	
	OR	%95 CI	OR	%95 CI	OR	%95 CI	OR	%95 CI	OR	%95 CI
**Short axis**	2.05	0.72–5.84	1.34	0.42–4.3	1.16	0.29–4.69	2.09	0.56–7.83	1.28	0.18–9.35
**Axis ratio**	3.38	1.44–9.9	3.94	1.38–11.27	3.59	1.07–12.07	2.61	0.87–7.88	2.81	0.68–11.61
**Hilum**	5.21	1.96–13.84	3.63	1.26–10.48	6.5	1.83–23.01	4.68	1.31–16.75	11.69	1.9–71.75
CDUS*			5.83	2.2–15.47	1.73	0.57–5.22	1.71	0.56–5.28	0.58	0.12–2.78
pSWE*					16.1	4.93–52.22			36.91	6.55–207.9
ADC*							3.55	1.12–11.21	11.48	2.09–62.77
**Sensitivity**	%79.4		%84.1		%87.3		%75		%90.9	
**Specificity**	%71.7		%73.6		%83		%70.7		%80.5	
**PPV**	%76.9		%79.1		%85.9		%73.3		%83.3	
**NPV**	%74.5		%79.6		%84.6		%72.5		%89.2	
**Accuracy**	%75.9		%79.3		%85.3		%72.9		%85.9	
**Nagelkerke R** **^2^**	0.382		0.439		0.625		0.437		0.682	
**Hosmer & Lemeshow**	0.780		0.268		0.374		0.548		0.500	

ADC: Apparent Diffusion Coefficient, CDUS: Color Doppler Ultrasound, CI: Confidence Interval, NPV: Negative Predictive Value, OR: Odds Ratio, PPV: Positive Predictive Value, pSWE: Point Shear Wave Elastography.
